# Endocarditis by Streptococcus pasteurianus

**DOI:** 10.7759/cureus.34529

**Published:** 2023-02-02

**Authors:** Catarina Pereira, Fernando Nogueira, José Cunha Marques, José Pestana Ferreira, Jorge S Almeida

**Affiliations:** 1 Internal Medicine, Centro Hospitalar e Universitário de São João, Porto, PRT; 2 Medicine, Faculdade de Medicina da Universidade do Porto (FMUP), Porto, PRT; 3 Internal Medicine, Centro Hospitalar Universitário de São João, Porto, PRT

**Keywords:** streptococcus pasteurianus, streptococcus gallolyticus, endocarditis, cytopenia, blood culture

## Abstract

The diagnosis of infective endocarditis is challenging because it has a variable clinical presentation and nonspecific symptoms and can present in different forms, especially when an unusual etiological agent is involved. We present the case of a female in her 70s admitted to the hospital with a medical history of bicytopenia, severe aortic stenosis, and rheumatoid arthritis. She had several consultations during which she presented with asthenia and general malaise. A septic screen test was performed that would determine that *Streptococcus pasteurianus* was present in a blood culture (BC), which was not valued. About three months later, she was hospitalized. In the first 24 hours of admission, the septic screen test was repeated and *Streptococcus pasteurianus* was isolated in BC. Splenic infarctions and transthoracic echocardiography suggested probable endocarditis, which was confirmed with transesophageal echocardiography. She underwent surgical intervention to remove the perivalvular abscess and replace the aortic prosthesis.

## Introduction

Endocarditis is one of the most challenging diagnoses in medicine because of its variable clinical presentation and different severities associated with nonspecific symptoms but with fever as the most common. An early diagnosis is essential for a favorable outcome, and a delay in diagnosis is often associated with more valvular complications and embolization of other organs. Infective endocarditis is associated with multiple complications, namely neurologic, cardiac, and septic emboli; immune reactions; and metastatic infection. Additionally, infective endocarditis has high mortality and morbidity, and despite improving diagnostic and treatment tools, the incidence is rising worldwide [1-5].

The diagnosis is based on clinical presentation, positive blood cultures (BC), and echocardiography (transthoracic or transesophageal). Modified Duke criteria is a helpful tool for diagnosis that includes clinical and pathologic criteria with primary (positive BC for infective endocarditis such as typical microorganisms or persistently positive BCs and evidence of endocardial involvement) and secondary criteria (such as fever, predisposition for this infection, microbiologic evidence, and vascular or immunologic phenomena) [6].

This case reinforces the need for high clinical suspicion in the face of a nonspecific, insidious, and relapsing clinic. It supports the importance of microbiological findings and their contextualization in the clinic, particularly when a microbial agent (like *S. pasteurianus*) is rarely described in the literature.

## Case presentation

We present the case of a female in her 70s with a history of severe aortic stenosis, who underwent valve replacement surgery approximately 1.5 years ago and had a biological prosthesis in the aortic position (functioning in the last transthoracic echocardiogram). The previous history also showed relevant chronic immune thrombocytopenia, followed by a clinical hematology consultation, diagnosed about nine years ago and treated with corticosteroids and eltrombopag. Thrombocytopenia is initially interpreted as having an association with valvular disease, but after cardiac surgery, the patient’s platelet values had not improved. At this point, a broad study was conducted to determine the etiology of thrombocytopenia. A myelogram revealed that the megakaryocytic series was hyperplastic with dysmorphic elements, focally in a peritrabecular location, forming nests and moderate reinforcement (MF2) of the reticulin network. Because of bone marrow with more evident myelodysplasia lesions in the megakaryocytic series, we then believed it was a primary medullary disease (myelodysplastic syndrome or myelodysplasia/megakaryocytic dysplasia) versus reactive alterations in a patient receiving eltrombopag. She had complained of generalized polyarthralgia and analytically presented a progressive increase in rheumatoid factor, so she was referred to a rheumatologist because of the possibility of rheumatoid arthritis. Analytically, the rheumatoid factor kept the ascending profile. Before admission, she was prescribed eltrombopag 75 mg daily and deflazacort 30 mg on alternate days.

Because of general malaise, asthenia, weight loss (6 kg in the last month), nocturnal hyper sweating, and daily fever peaks (predominantly in the morning and with a partial resolution with antipyretics), she went to the emergency department. Physical examination showed a poor general condition, mucocutaneous pallor, palatine petechiae and lateral region of the tongue, and low blood pressure (93/43 mmHg), with no other alterations without palpable adenopathies. The chest X-ray showed no evidence of infiltrates or consolidations. Analytically, she had mild microcytic hypochromic anemia (Hb 10.2 g/dL), leukocytosis of 14.97x10^9^/L (with mild neutrophilia and lymphopenia), and severe thrombocytopenia with platelet counts less than 10x10^9^/L, an elevation of lactate dehydrogenase 334 U/L and C-reactive protein 310.7 mg/L (normal reference value < 3 mg/L), with normal renal function. Fluid therapy was started, and the corticosteroid dose was adjusted to 1mg/kg/day.

**Figure 1:**
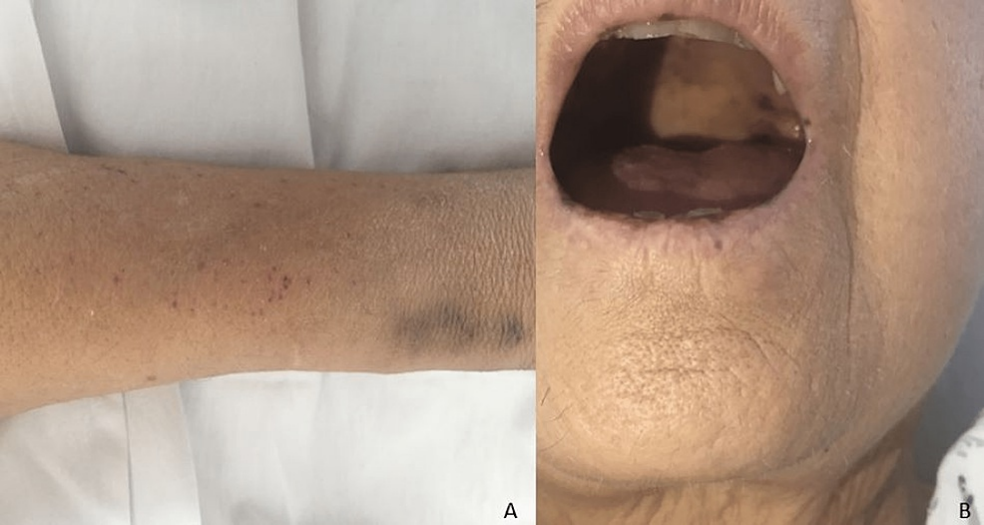
Patient images at the time of admission Ecchymosis and petechiae on the left arm (A) and petechiae on the palate and tongue (B).

Blood, urine, and sputum were collected for a microbiological study, and she was admitted to the Internal Medicine department. On admission, there were additional findings: discolored and dehydrated skin and mucous membranes, scattered ecchymosis on both upper limbs (Figure 1A), and petechiae on the palate and tongue (Figure 1B). In hemodynamic terms, she had low blood pressure (96/47 mmHg). On auscultation, she had a systolic murmur, grade II/VI, and signs of pulmonary and peripheral congestion with moderate swelling up to the knees bilaterally. She started empiric antibiotic therapy with piperacillin 4000 mg + tazobactam 500 mg intravenous t.i.d (thrice in a day).

In reviewing her past medical history, it was found that approximately four months before admission, in the clinical hematology consultation, the patient had already described complaints of asthenia, generalized malaise, dysuria, and urinary frequency with three days of evolution without mentioning any other complaints such as fever. Analytically, she had hypochromic microcytic anemia (hemoglobin 10 g/dL), leukocytosis 21.49x10^9^/L with associated neutrophilia, thrombocytopenia 31x10^9^/L, and elevated C-reactive protein 103.4 mg/L. Urinalysis showed leukocyturia of 1251/uL (normal reference value < 30/uL). Blood and urine cultures were collected. The patient completed one week of empirical antibiotic therapy with ciprofloxacin 500 mg bid. A strain of multi-sensitive *Streptococcus pasteurianus* was isolated only in one of the two BCs, and in the microbiological examination of the urine, *Candida albicans* was isolated.

About two weeks later, there was a clinical improvement with a progressive reduction of inflammatory parameters. About a month later, at the follow-up hematology consultation, she had a recrudescence in the inflammatory parameters with a significant increase in C-reactive protein (114.5 mg/L). However, she remained clinically asymptomatic and without any clinical focus on infection.

Two months before admission, the patient went to the emergency department, complaining of epigastric abdominal pain with several weeks of evolution, nausea, dysphagia, weight loss of about 6 kg in the past few days, asthenia, and anorexia, and she had recorded a fever peak on the previous day of 38°C. On physical examination, she had aortic systolic murmur III/VI, jaundice, and oropharyngeal candidiasis. Analytically, she had bicytopenia superimposed on her normal values (hemoglobin 10 g/dL and 40x10^9^/L platelets), de novo hyperbilirubinemia with total bilirubin 2.44 mg/dL, and elevation of lactate dehydrogenase 255 U/L. She underwent an abdominal ultrasound, which revealed splenomegaly with a spleen of 14.4 cm in diameter. An upper gastrointestinal endoscopy was performed, and it provided evidence of extensive esophageal candidiasis. She was treated with fluconazole and esomeprazole, maintaining chronic corticosteroid therapy at a dose of 30 mg deflazacort every other day and eltrombopag 75 mg qd (once daily).

In the bacteriological exams of blood collected at the hospital, a multi-sensitive strain of* Streptococcus pasteurianus *was isolated early at 24 hours of culture. The remaining microbiological study was negative. On the second day of hospitalization, a computed tomography scan was performed, revealing the presence of multiple splenic infarctions. A transesophageal echocardiogram revealed thickening of the leaflets; an extensive vegetating mass at the level of the left coronary leaflet with vegetation measuring 24x5 mm; a periprosthetic abscess involving half of the circumference of the ring (between 11-05h); and multiseptate without prosthetic obstruction (mean transvalvular pressure gradient <20 mmHg) and with mild prosthetic regurgitation without other alterations. The presumptive diagnosis of prosthetic valve endocarditis was assumed, and the antibiotic therapy was adjusted to ceftriaxone 2 mg qd plus gentamicin 160 mg qd intravenously; surgical intervention was scheduled.

The initial hypothesis was that it was an autoimmune disease flare-up because the patient presented a clinical history of arthralgias, nonspecific constitutional symptoms, and an elevation of the rheumatoid factor, the patient was already under chronic corticosteroid therapy without any improvement. Additionally, given the constitutional picture presented, with a subacute/chronic evolution, the possibility of it being a neoplastic, solid organ or hematological condition was also considered.

On the 15th day of hospitalization, surgical intervention was performed with the excision of the perivalvular abscess with a heterologous pericardium flap and replacement of the aortic bioprosthesis by another bioprosthesis. The intraoperative transesophageal echocardiogram showed no evidence of complications, as did the transthoracic echocardiogram. The microbiological culture of the bioprosthetic valve was surprisingly negative. She completed six weeks of antibiotic therapy, having already finished the course of antibiotics during home hospitalization while maintaining sustained clinical, hemodynamic, and apyrexia stability. After finishing treatment, the patient showed a sustained improvement in inflammatory parameters and clinical stability. She showed resolution of the initial symptoms. She was referred to her attending physician for further follow-up and endoscopic study. Hematology, infectious diseases, and cardiothoracic surgery also followed her case.

## Discussion

Several agents can cause endocarditis, with the most frequent being *Staphylococcus aureus, Streptococci of the Viridans group, Staphylococcus gallolyticus*, and the *HACEK group* (*Haemophilus parainfluenzae, H. aphrophilus, H. paraphrophilus, H. influenzae, Actinobacillus actinomycetemcomitans, Cardiobacterium hominis, Eikenella corrodens, Kingella kingae, and K. denitrificans*) [7]. *Streptococcus pasteurianus* is a subspecies of *Streptococcus gallolyticus*, belonging to *group D Streptococcus*, which integrates the normal oral and gastrointestinal flora in children and adults and may be a potential pathogen. Epidemiologically, it has a higher incidence in newborns and the elderly, with some preference for males [8,9]. It is an uncommon agent and rarely mentioned in the literature that is infrequently associated with endocarditis. However, when described as the etiologic agent of endocarditis, it is usually associated with large vegetations, with a potentially greater risk of embolization and the need for surgical intervention.

Regarding the clinical presentation, it tends to present in a subacute way. When isolated in BCs, colon neoplasms should be actively investigated, particularly in the case of bacteremia associated with endocarditis, with colonoscopy assuming a vital role in the early detection of lesions. 

In the case presented, it was an elderly woman, with several factors of immunosuppression, namely autoimmune disease, therapy with corticosteroids, and on the other hand, risk factors for endocarditis, particularly the fact that she had a prosthetic valve. The nonspecific symptomatology and the initial improvement after the first courses of antibiotics (in the context of a urinary tract infection) delayed the assessment of blood cultures and the final diagnosis. A high index of suspicion is fundamental in the diagnosis of endocarditis, and applying the modified Duke criteria is a particularly useful diagnostic tool in the face of suspicion.

Given positive blood cultures with isolation of a subspecies of *Streptococcus gallolyticus* together with echocardiographic findings suggestive of endocarditis (extensive vegetation and periprosthetic abscess) the diagnosis of infective endocarditis was made.

The choice of antibiotic regimen with ceftriaxone and gentamicin followed international recommendations, namely European guidelines [7]. As it involved a large amount of vegetation (<15mm), the case deserved a multidisciplinary discussion, namely with cardiac surgeons, opting for the decision of surgical intervention to remove the abscess.

## Conclusions

*Streptococcus gallolyticus *subspecies* pasteurianus* is a cause and a potential pathogen of bacteremia, infective endocarditis, and urinary tract infection in elderly and immunodeficient people. *Streptococcus pasteurianus* is assumed to be a rare agent with a high potential for seriousness, and screening is recommended.

This case revealed an indolent presentation of endocarditis, with months of evolution from the first microbiological isolation to the positive diagnosis of endocarditis, in a patient with chronic immunosuppression and some confounding factors that somehow hindered and delayed the diagnosis. Despite the indolent clinical manifestation, the patient presented a significant valvular involvement with important local complications and distant embolization, thus alerting her to the potential severity of* S. pasteurianus* infection despite its rarity.
